# Diagnosis of Malarial Diathesis

**Published:** 1863-02

**Authors:** 


*Diagnosis of Malarial Diathesis—New Test Symptom.*—Our contemporary of the *Boston Medical & Surgical Journal*, publishes a paper placed in the editor's hands by Surgeon General Dale, of Mass., to whom it was communicated by Surgeon E. C. Bidwell, of the 31st Mass. Volunteers. This paper purports to give "a *new pathognomonic symptom of the malarial diathesis.*"

After alluding to the frequent difficulty in diagnosis in these cases, and nevertheless its importance, he describes the symptoms:

It is a very peculiar and abnormal appearance of the tongue, in which its under surface appears to have trespassed upon the upper, the *papillæ* of the latter being supplanted by the transverse *rugæ* of the former. The sides are thickened and rounded, the normal well-defined edge being obliterated, and the line of demarcation moved nearer to the mesial line. This appearance of the sides may be associated with any and every possible appearance of the remaining papillary surface, clean or coated, thick or thin, light or dark, just as the malarious disease may be attended by any and every variety of morbid condition of the system. Through all this variety it is perfectly distinct, and, when once learned by actual inspection, is unmistakeable.

Wherever I find this malarious tongue, whatever other symptoms and conditions may be present, I give quinine freely and confidently. So much confidence do I place in its indications that, when it is present in complicated or obscure cases, I make all other treatment wait upon quinine as the first and indispensable remedy. On the other hand, when in charge of a regiment, if soldiers came to me complaining of "chills," whose tongues did not present this appearance—telling the truth to the eye while they lied to the ear—they were unhesitatingly returned to duty. I am confident that in judging by this criterion of more than twelve hundred cases during the last season, I have never wronged more than two men, and those not grievously. In those two cases, I am obliged to confess, it was but faintly developed. Both were mild cases of simple intermittent.

We call our contemporary's attention to the fact that this is by no means a *new* symptom, as it most certainly is not a pathognomonic one. In a communication to the *Western Jour. Med. & Surg.*, Aug. 1851, (reprinted in the *American Journal of Med. Sciences*, Oct., 1851, p. 555), Dr. Thomas C. Osborne, of Erie, Ala., points out this same symptom. We extract a single paragraph from his article with regard to what he designates, "the *malarial margin*:"

"As the name imports, it is an essential departure from the normal aspect of the edge, constituting a distinct lateral boundary of the tongue, occupying more or less surface, according to the charge of infection in the system. Ordinarily, the color amounts only to a very faint bluish tinge, which is liable to be lost, or merged in the various tints imparted to the tongue in various diseases. The most fixed condition of this symptom is an appearance of indentation or crimpling transversely, which is apparently confined to the subjacent tissue, while the superficial integument is moist, smooth, and transparent. In a word, it seems to be a continuation or encroachment of the inferior surface upon the superior and lateral borders of the tongue, greater as we approach the root of that organ."

Dr. Osborne attributed great importance to this condition of the tongue as proving "malarious," poisoning, and as indicating the use of quinine. He asserted that he was able, by observing it, to predict an attack of malarious disease, many days before its occurrence.

Largely familiar as we have been with the multiform varieties of intermittent and remittent, we must be permitted to say, that although this condition of the tongue is quite common in these forms of disease, yet it is by no means constant, even in the most strongly marked cases. In these, as elsewhere, the tongue is prone to deceive. It is an occasional, but not a pathognomonic symptom.

---

*The American Medical Association.*—There is a vague rumor that the Committee of Arrangements or some others in authority propose to call a meeting of the American Medical Association the ensuing season. We fervently trust no such stupendous folly will find birth at present. The reasons previously existing for non-assemblage of that body are tenfold more powerful now than at any time during the previous two years. The matter is beyond argument. Do the gentlemen of the Committee propose to recognize secession as a *fait accompli?* Or do they propose that the fragment shall assume to be the whole? There is no conceivable reason why the Association should be called together, unless perhaps to familiarize the professional as well as the public mind with the idea of a divided nation. We repeat we trust no such absurdity as rumor indicates will be perpetrated by the Committee. Let the State and other local societies be fully sustained, but let the national representative body of the profession wait for return of the halcyon times of peace.

